# A new pendulum motion with a suspended point near infinity

**DOI:** 10.1038/s41598-021-92646-6

**Published:** 2021-06-24

**Authors:** A. I. Ismail

**Affiliations:** 1 Mechanical Engineering Department, College of Engineering and Islamic Architecture, Umm Al-Qura University, P. O. Box 5555, Mecca, Saudi Arabia; 2 Mathematics Department, Faculty of Science, Tanta University, P. O. Box 31527, Tanta, Egypt

**Keywords:** Mechanical engineering, Applied mathematics, Applied physics

## Abstract

In this paper, a pendulum model is represented by a mechanical system that consists of a simple pendulum suspended on a spring, which is permitted oscillations in a plane. The point of suspension moves in a circular path of the radius (a) which is sufficiently large. There are two degrees of freedom for describing the motion named; the angular displacement of the pendulum and the extension of the spring. The equations of motion in terms of the generalized coordinates $\varphi$ and $\xi$ are obtained using Lagrange’s equation. The approximated solutions of these equations are achieved up to the third order of approximation in terms of a large parameter $\varepsilon$ will be defined instead of a small one in previous studies. The influences of parameters of the system on the motion are obtained using a computerized program. The computerized studies obtained show the accuracy of the used methods through graphical representations.

## Introduction

The pendulum motions are studied in many works^1–7^. The motion of the pendulum on an ellipse is studied in^8^. The supported point of this pendulum moves on an ellipse path while the end moves with arbitrary angular displacements. The equation of motion is obtained and solved for one degree of freedom $\varphi$. In^9^, the relative periodic solutions of a rigid body suspended on an elastic string in a vertical plane are considered. The equations of motion are obtained and solved in terms of the small parameter $\varepsilon$. Computerized data and graphical representations of the solutions are obtained for describing the behavior of the pendulum of some periods. The spherical pendulum motion with an arbitrary three-dimensional periodic vibration of the suspension point is considered in^10^. The controlling parameters of suspension point vibrations necessary for pendulum stabilization at a set point on the sphere are found. The stable model of oscillations at which the root mean square velocity of the suspension point vibrations is lowest was investigated. The axisymmetric oscillations of a spherical pendulum with vertical suspension point vibration were studied in^11^. The problem of determining stable periodic solutions for high-frequency suspension point vibrations is reduced to the condition of a minimum of the effective potential energy in^12–14^. The cases of the harmonically excited, damped, and external forces of spring pendulum models are considered in^15–17^ for different mechanical systems. The motion in a fluid flow for vibrated spring pendulum is considered in^18^. The dynamical and vibrational behaviors of the rigid body pendulum are considered in^19,20^.

## Formulation of the problem

**Figure 1:**
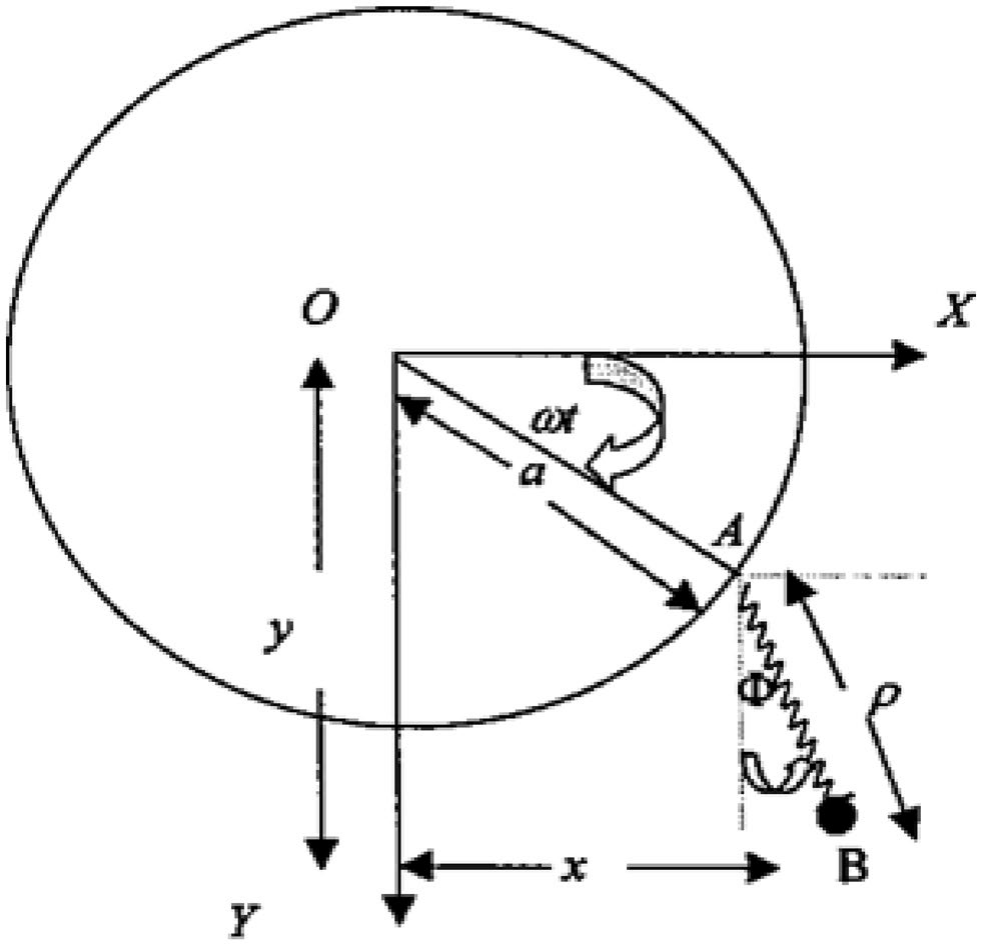
The pendulum motion on a sufficiently large circle.

Consider a pendulum of unit mass^21^ located at point *B(x,y)*, suspended on a linear massless elastic spring of length $\rho$ with supported point *A* moving on a circle of radius (a $\to \infty$) with constant angular velocity $\omega$ in an anticlockwise direction (see Fig. 1). If the motion starts at *t* = 0 when the spring coincides with OX*,* then after time *t*, reaches point *A* and the string makes the angle $\Phi$ with *Y-*axis, and the radius *OA* makes an angle $\omega {\kern 1pt} t{\kern 1pt} \,$ with *X-*axis, so we write:

The kinetic energy:

1 $$\begin{aligned} T & = \frac{1}{2}[( - a\omega \sin \omega t + \rho \dot{\Phi }\cos \Phi + \dot{\rho }\sin \Phi )^{2} + ( - a\omega \cos \omega t - \rho \dot{\Phi }\sin \Phi + \dot{\rho }\cos \Phi )^{2} ] \\ & = \frac{1}{2}[a^{2} \omega ^{2} + \dot{\rho }^{2} + \rho ^{2} \dot{\Phi }^{2} - 2a\rho \omega \dot{\Phi }\sin (\Phi - \omega t) - 2a\dot{\rho }\omega \cos (\Phi - \omega t)]. \\ \end{aligned}$$

The potential energy is expressed as:

2 $$V = \frac{1}{2}k^{2} (\rho - b)^{2} - \rho g\cos \Phi ,$$

where $k^{2}$ is the elastic constant, b is the string's length at rest and g is the gravitational attraction. Consider the parameters and variables:

3 $$\begin{aligned} \varepsilon & = \frac{a}{b} \succ \succ 1,\quad i.e.\quad a \succ \succ b,\quad \omega _{n}^{2} = \frac{g}{b}, \\ \Omega & = \frac{{\omega _{n} }}{\omega },\quad \Phi = \omega \varphi ,\quad \tau = \omega t, \\ \end{aligned}$$

where $\varepsilon$ is the large parameter and $\omega _{n}$ is the natural angular velocity. Substituting from (3) into (1) and (2), one has:

4 $$T = 0.5\,[\varepsilon ^{{ - 2}} b^{2} \omega ^{2} + \varepsilon ^{{ - 2}} \omega ^{2} \rho ^{2} \varphi ^{{'2}} + \omega ^{2} \rho ^{{'2}} + 2\varepsilon ^{{ - 2}} \omega ^{2} b\rho \,\varphi ^{\prime}\sin (\varepsilon ^{{ - 1}} \varphi - \tau ) - b\,\varepsilon ^{{ - 1}} \omega ^{2} \rho ^{\prime}\cos (\varepsilon ^{{ - 1}} \varphi - \tau )],$$

5 $$V = \frac{1}{2}k^{2} (\rho - b)^{2} - b\omega ^{2} \Omega ^{2} \rho \cos (\varepsilon ^{{ - 1}} \varphi ),$$

where the primes denote the derivative for $\tau$. The Lagrangian function *L* is defined as:

$${\text{L}}\, = \,{\text{T}} - {\text{V}}.$$

Using (4) and (5) we get:

6 $$\begin{aligned} L & = 0.5\omega ^{2} [\varepsilon ^{{ - 2}} b^{2} + \rho ^{{'2}} + \rho ^{2} \varepsilon ^{{ - 2}} \varphi ^{{'2}} + 2\varepsilon ^{{ - 2}} b\rho \varphi ^{\prime}(\sin \varepsilon ^{{ - 1}} \varphi \cos \tau - \sin \varepsilon ^{{ - 1}} \varphi \sin \tau ) \\ & \quad \quad \quad \quad \,\,\,\,\, - 2\varepsilon ^{{ - 1}} b\rho ^{\prime}(\cos \varepsilon ^{{ - 1}} \varphi \cos \tau + \sin \varepsilon ^{{ - 1}} \varphi \sin \tau ) + 2b\Omega ^{2} \rho \cos \varepsilon ^{{ - 1}} \varphi - k^{2} (\rho - b)^{2} /\omega ^{2} ]. \\ \end{aligned}$$

Making use of the following expansions:

7 $$\begin{aligned} \sin \varepsilon ^{{ - 1}} \varphi & = \varepsilon ^{{ - 1}} \varphi - \frac{{\varepsilon ^{{ - 3}} \varphi ^{3} }}{{3!}} + \frac{{\varepsilon ^{{ - 5}} \varphi ^{5} }}{{5!}} - \cdots , \\ \cos \varepsilon ^{{ - 1}} \varphi & = 1 - \frac{{\varepsilon ^{{ - 2}} \varphi ^{2} }}{{2!}} + \frac{{\varepsilon ^{{ - 4}} \varphi ^{4} }}{{4!}} - \cdots . \\ \end{aligned}$$

Equation (6) up to the third approximation, neglecting terms of the order $\varepsilon ^{{ - 4}}$, becomes:

8 $$\begin{aligned} L & = 0.5\,\omega ^{2} [\varepsilon ^{{ - 2}} b^{2} + \rho ^{{'2}} + \rho ^{2} \varepsilon ^{{ - 2}} \varphi ^{{'2}} + 2\varepsilon ^{{ - 3}} b\rho \varphi ^{\prime}\varphi \cos \tau \\ & \quad \quad \quad \quad - 2\varepsilon ^{{ - 2}} b\varphi ^{\prime}\sin \tau - \varepsilon ^{{ - 1}} b\rho ^{\prime}(2 - \varepsilon ^{{ - 2}} \varphi ^{2} )\cos \tau \\ & \quad \quad \quad \quad - 2\varepsilon ^{{ - 2}} b\rho ^{\prime}\varphi \sin \tau + b\Omega ^{2} \rho (2 - \varepsilon ^{{ - 2}} \varphi ^{2} ) - k^{2} (\rho - b)^{2} /\omega ^{2} ]. \\ \end{aligned}$$

## Equations of motion

For our model, Lagrange's equations^22^ take the form,

9 $$\begin{gathered} \frac{d}{{d\tau }}\frac{{\partial L}}{{\partial \rho ^{\prime}}} - \frac{{\partial L}}{{\partial \rho }} = 0, \hfill \\ \frac{d}{{d\tau }}\frac{{\partial L}}{{\partial \varphi ^{\prime}}} - \frac{{\partial L}}{{\partial \varphi }} = 0, \hfill \\ \end{gathered}$$

where $\rho ,\;\varphi$ and $\rho ^{\prime},\;\varphi ^{\prime}$ represent the generalized coordinates and generalized velocities, respectively. Making use of (8), one gets:

$$\begin{aligned} \frac{{\partial L}}{{\partial \varphi ^{\prime}}} & = \omega ^{2} \rho \varepsilon ^{{ - 2}} (\rho \,\varphi ^{\prime} - b\sin \tau + \varepsilon ^{{ - 1}} b\varphi \cos \tau ), \\ \frac{{\partial L}}{{\partial \varphi }} & = - \varepsilon ^{{ - 2}} \omega ^{2} b[\rho ^{\prime}\sin \tau + \Omega ^{2} \rho \varphi - \varepsilon ^{{ - 1}} \cos \tau (\rho \varphi ^{\prime} + \rho ^{\prime}\varphi )], \\ \frac{{\partial L}}{{\partial \rho ^{\prime}}} & = b\omega ^{2} [\rho ^{\prime}/b - \varepsilon ^{{ - 1}} \cos \tau - \varepsilon ^{{ - 2}} \varphi \sin \tau + 0.5\varepsilon ^{{ - 3}} \varphi ^{2} \cos \tau ], \\ \frac{{\partial L}}{{\partial \rho }} & = \omega ^{2} [b\Omega ^{2} - k^{2} (\rho - b)/\omega ^{2} + \varepsilon ^{{ - 2}} (\rho \varphi ^{{'2}} - b\varphi ^{\prime}\sin \tau - 0.5\Omega ^{2} b\,\varphi ^{2} + \varepsilon ^{{ - 1}} b\varphi \varphi ^{\prime}\cos \tau )]. \\ \end{aligned}$$

So, using (9), we write:

10 $$\rho \varphi ^{\prime\prime} + 2\rho ^{\prime}\varphi ^{\prime} - \varepsilon ^{{ - 1}} b\varphi \sin \tau - b\cos \tau + b\Omega ^{2} \varphi = 0,$$

11 $$\begin{aligned} & \rho ^{\prime\prime} - b\,\Omega ^{2} + k^{2} (\rho - b)/\omega ^{2} + \varepsilon ^{{ - 1}} b\sin \tau - \varepsilon ^{{ - 2}} (b\,\varphi \cos \tau - \rho \,\varphi ^{{'2}} \\ & \quad \quad + 0.5\,\Omega ^{2} b\,\varphi ^{2} ) - 0.5\,\varepsilon ^{{ - 3}} b\,\varphi ^{2} \sin \tau = 0. \\ \end{aligned}$$

Consider:

12 $$\rho = b + \xi (\tau ),\quad k^{2} /\omega ^{2} = \sigma ^{2}$$

where $\xi$ is function of the new variable $\tau$, then Eqs. (10) and (11) become:

13 $$\varphi ^{\prime\prime} + \Omega ^{2} \varphi = \cos \tau - (\xi \varphi ^{\prime\prime} + 2\xi ^{\prime}\varphi ^{\prime})/b + \varepsilon ^{{ - 1}} \varphi \sin \tau ,$$

14 $$\begin{aligned} \xi ^{\prime\prime} + \sigma ^{2} \xi & = b\,\Omega ^{2} - \varepsilon ^{{ - 1}} b\sin \tau + \varepsilon ^{{ - 2}} (b\varphi \cos \tau + b\varphi ^{{'2}} + \xi \varphi ^{{'2}} \\ & \quad - 0.5\,\Omega ^{2} b\,\varphi ^{2} ) + 0.5\varepsilon ^{{ - 3}} b\varphi ^{2} \sin \tau . \\ \end{aligned}$$

Equations (13) and (14) are the equations of motion and represent a quasilinear system of second-order to be solved in terms of the generalized coordinates $\xi$ and $\varphi$ using the large parameter method. The expressions of $\varphi$ and $\xi$ are expected to be functions of $\varepsilon$ and depend on the values of $\Omega$ and $\sigma$*.* In other words if $\Omega$ and $\sigma \ne 1,2,3,...$ are integer values, the resonance case of oscillations is obtained, while if $\Omega$ and $\sigma$ are non-integers the non-resonance case or the fundamental oscillations of the system is obtained.

## Approximate periodic solutions

To find the perturbed solutions for non-resonance cases up to the third approximation, we use the method of large parameter^23–25^. So we seek these solutions in the form:

15 $$\begin{aligned} \varphi (\varepsilon ,\tau ) & = \varphi _{0} (\tau ) + \varepsilon ^{{ - 1}} \varphi _{1} (\tau ) + \varepsilon ^{{ - 2}} \varphi _{2} (\tau ) + \varepsilon ^{{ - 3}} \varphi _{3} (\tau ) + \cdots , \\ \xi (\varepsilon ,\tau ) & = \xi _{0} (\tau ) + \varepsilon ^{{ - 1}} \xi _{1} (\tau ) + \varepsilon ^{{ - 2}} \xi _{2} (\tau ) + \varepsilon ^{{ - 3}} \xi _{3} (\tau ) + \cdots , \\ \end{aligned}$$

Substituting from Eqs. (15) into Eqs. (13) and (14) respectively, then equating coefficients of like powers of $\varepsilon$ in both sides, we have:

16 $$\varphi ^{\prime\prime}_{0} + \Omega ^{2} \varphi _{0} = \cos \tau - (\xi _{0} \varphi ^{\prime\prime}_{0} + 2\xi ^{\prime}_{0} \varphi ^{\prime}_{0} )/b,$$

17 $$\varphi ^{\prime\prime}_{1} + \Omega ^{2} \varphi _{1} = \varphi _{0} \sin \tau - (\xi _{0} \varphi ^{\prime\prime}_{1} + \xi _{1} \varphi ^{\prime\prime}_{0} + 2\xi ^{\prime}_{0} \varphi ^{\prime}_{1} + 2\xi ^{\prime}_{1} \varphi ^{\prime}_{0} )/b,$$

18 $$\varphi ^{\prime\prime}_{2} + \Omega ^{2} \varphi _{2} = \varphi _{1} \sin \tau - (\xi _{0} \varphi ^{\prime\prime}_{2} + \xi _{1} \varphi ^{\prime\prime}_{1} + \xi _{2} \varphi ^{\prime\prime}_{0} + 2\xi ^{\prime}_{0} \varphi ^{\prime}_{2} + 2\xi ^{\prime}_{1} \varphi ^{\prime}_{1} + 2\xi ^{\prime}_{2} \varphi ^{\prime}_{0} )/b,$$

19 $$\varphi ^{\prime\prime}_{3} + \Omega ^{2} \varphi _{3} = \varphi _{2} \sin \tau - (\xi _{0} \varphi ^{\prime\prime}_{3} + \xi _{1} \varphi ^{\prime\prime}_{2} + \xi _{2} \varphi ^{\prime\prime}_{1} + \xi _{3} \varphi ^{\prime\prime}_{0} + 2\xi ^{\prime}_{0} \varphi ^{\prime}_{3} + 2\xi ^{\prime}_{1} \varphi ^{\prime}_{2} + 2\xi ^{\prime}_{2} \varphi ^{\prime}_{1} + 2\xi ^{\prime}_{3} \varphi ^{\prime}_{0} )/b,$$

20 $$\xi ^{\prime\prime}_{0} + \sigma ^{2} \xi _{0} = b\Omega ^{2}$$

21 $$\zeta ^{\prime\prime}_{1} + \sigma ^{2} \zeta _{1} = - b\sin \tau$$

22 $$\xi ^{\prime\prime}_{2} + \sigma ^{2} \xi _{2} = b\varphi _{0} \cos \tau + b\varphi _{0} ^{{'2}} + \xi _{0} \varphi _{0} ^{{'2}} - 0.5\,\Omega ^{2} b\varphi _{0}^{2} ,$$

23 $$\xi ^{\prime\prime}_{3} + \sigma ^{2} \xi _{3} = b\varphi _{1} \cos \tau + 2b\varphi ^{\prime}_{0} \varphi ^{\prime}_{1} + 2\xi _{0} \varphi ^{\prime}_{0} \varphi ^{\prime}_{1} + \xi _{1} \varphi _{0}^{{'2}} - \Omega ^{2} b\varphi _{0} \varphi _{1} + 0.5b\varphi _{0}^{2} \sin \tau .$$

Using the principle of superposition^26,27^, we neglect the complementary part of the solutions of the differential Eqs. (16)–(23), so that the particular solutions are obtained as:

24 $$\varphi _{0} = A\cos \tau ,$$

25 $$\varphi _{1} = AB\sin 2\tau ,$$

26 $$\varphi _{2} = A(G\cos 3\tau + H\cos \tau ),$$

27 $$\varphi _{3} = A(I\sin 4\tau + J\sin 2\tau ),$$

28 $$\xi _{0} = g/\omega ^{2} \sigma ^{2} ,$$

29 $$\xi _{1} = - b\sin \tau /\sigma ^{2} - 1,$$

30 $$\xi _{2} = bC + bD\cos 2\tau ,$$

31 $$\xi _{3} = bAE\sin 3\tau + bAF\sin \tau ,$$

where

$$\begin{aligned} A & = \frac{{\gamma ^{2} }}{{\Omega ^{2} (\gamma ^{2} - 1)}},\,\,\,\,\,B = \frac{{\gamma ^{2} (\sigma ^{2} - 4)}}{{2\Omega ^{2} (\gamma ^{2} - 4)(\sigma ^{2} - 1)}},\,\,\,\,\,C = A\left( {\frac{{2\gamma ^{2} - A\Omega ^{2} \gamma ^{2} + 2A\Omega ^{2} }}{{4\gamma ^{2} }}} \right), \\ D & = \frac{A}{{2(\sigma ^{2} - 4)}}\left( {\frac{{2\gamma ^{2} - A\Omega ^{2} \gamma ^{2} - 2A\Omega ^{2} }}{{2\gamma ^{2} }}} \right),E = \frac{1}{{2(\sigma ^{2} - 9)}}\left[ {B - AB\Omega ^{2} + \frac{A}{{2(\sigma ^{2} - 1)}} + \frac{A}{4} - \frac{{4AB\Omega ^{2} }}{{\gamma ^{2} }}} \right], \\ F & = \frac{1}{{2(\sigma ^{2} - 1)}}\left[ {B - AB\Omega ^{2} - \frac{A}{{2(\sigma ^{2} - 1)}} + \frac{A}{4} + \frac{{4AB\Omega ^{2} }}{{\gamma ^{2} }} - \frac{A}{{(\sigma ^{2} - 1)}}} \right], \\ G & = \frac{1}{{2(\gamma ^{2} - 9)}}\left[ {\frac{{B\gamma ^{2} (\sigma ^{2} - 5)}}{{\Omega ^{2} (\sigma ^{2} - 1)}} - \frac{{3\gamma ^{2} D}}{{\Omega ^{2} }} + \frac{{4B}}{{(\sigma ^{2} - 1)}}} \right], \\ H & = \frac{1}{{2(\gamma ^{2} - 1)}}\left[ {\frac{{B\gamma ^{2} (5 - \sigma ^{2} )}}{{\Omega ^{2} (\sigma ^{2} - 1)}} + \frac{{5\gamma ^{2} D}}{{\Omega ^{2} }} + \frac{{4B}}{{(\sigma ^{2} - 1)}} + \frac{{2C\gamma ^{2} }}{{\Omega ^{2} }}} \right], \\ I & = \frac{1}{{2(\gamma ^{2} - 16)}}\left\{ {\frac{{G\gamma ^{2} (\sigma ^{2} - 16)}}{{\Omega ^{2} (\sigma ^{2} - 1)}} + \frac{{7\gamma ^{2} AE}}{{\Omega ^{2} }} + \frac{{12\gamma ^{2} BD}}{{\Omega ^{2} }}} \right\}, \\ J & = \frac{1}{{2(\gamma ^{2} - 4)}}\left\{ { - \frac{{\gamma ^{2} G(\sigma ^{2} - 4)}}{{\Omega ^{2} (\sigma ^{2} - 1)}} - \frac{{5\gamma ^{2} AE}}{{\Omega ^{2} }} + \frac{{\gamma ^{2} H(\sigma ^{2} - 4)}}{{\Omega ^{2} (\sigma ^{2} - 1)}} + \frac{{3\gamma ^{2} AF}}{{\Omega ^{2} }} + \frac{{\gamma ^{2} BC}}{{\Omega ^{2} }}} \right\}, \\ \gamma ^{2} & = b\Omega ^{2} /(9/k^{2} + b). \\ \end{aligned}$$

Substituting from (24)–(27) and (28)–(31) into (15), to obtain the following approximate periodic solutions in the form:

32 $$\begin{aligned} \varphi (\varepsilon ,\tau ) & = A[\cos \tau + \varepsilon ^{{ - 1}} B\sin 2\tau + \varepsilon ^{{ - 2}} (G\cos 3\tau + H\cos \tau ) \\ & \quad + \varepsilon ^{{ - 3}} (I\sin 4\tau + J\sin 2\tau )] + \cdots , \\ \end{aligned}$$

33 $$\begin{aligned} \xi (\varepsilon ,\tau ) & = \xi _{0} - b[\frac{{\varepsilon ^{{ - 1}} \sin \tau }}{{(\sigma ^{2} - 1)}} + \varepsilon ^{{ - 2}} (C + D\cos 2\tau ) \\ & \quad + \varepsilon ^{{ - 3}} A(E\sin 3\tau + F\sin \tau )] + \cdots , \\ \end{aligned}$$

where … indicate to terms of order lower than − 3.

## Graphical representations and numerical consideration

In this section, we investigate the graphical representations of the solutions (32) and (33) to describe the influence of the different parameters of the problem on the behavior of the motion. On the other hand, we achieved programs to obtain the solutions $(\varphi ,\tau )$, $(\xi ,\tau )$, and their stabilities $(\varphi ,\dot{\varphi })$ and $(\xi ,\dot{\xi })$. The characteristic curves of this motion are obtained when:

$$k^{2} = 4.30\,{\text{N}}/{\text{cm}},\quad g = 980\,{\text{cm}}/\text{s} ^{2} ,\quad a = 1\,{\text{cm}},\quad \omega = 2,\quad b = 20000\,{\text{cm}}.$$

**Figure 2:**
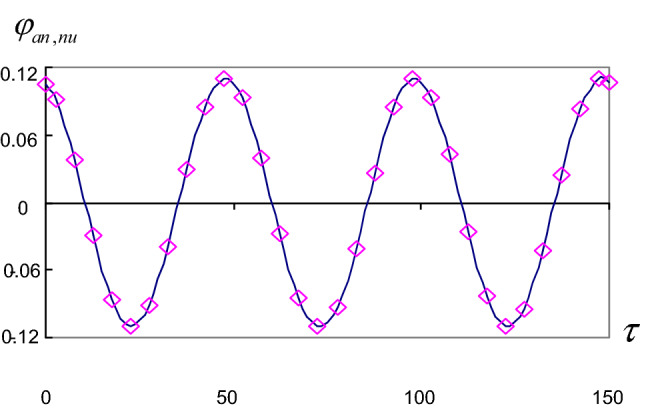
Approximate periodic solutions diagram ($\varphi _{{an,nu}}$, $\tau$).

**Figure 3:**
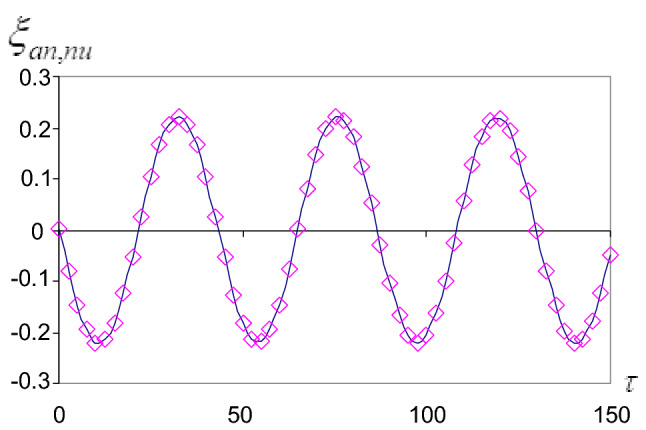
Approximate periodic solution diagram ($\xi _{{an,nu}}$, $\tau$).

**Figure 4:**
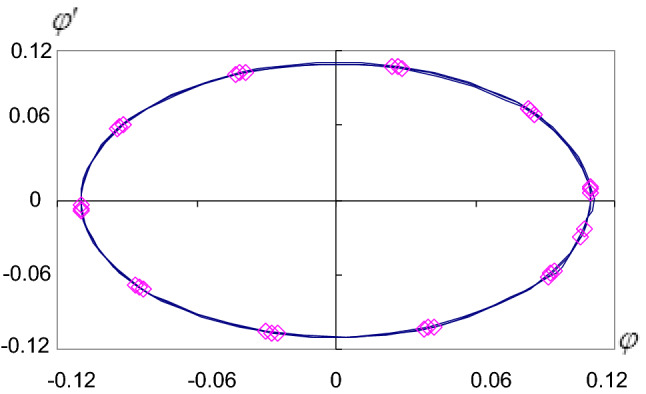
The stability diagram ($\varphi ^{\prime}$, $\varphi$).

**Figure 5:**
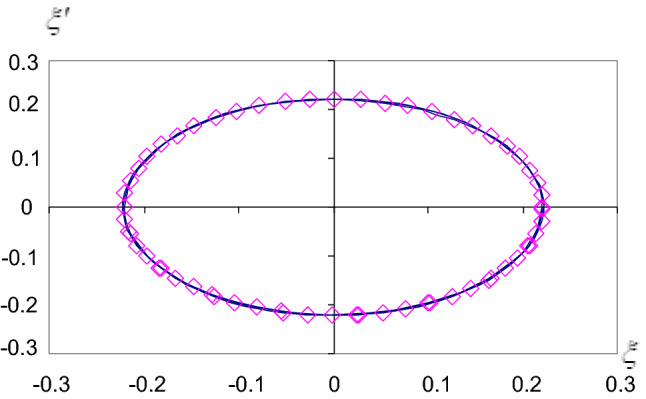
The stability diagram ($\xi ^{\prime},\xi$).

In each case, we calculate the constants A, B, C, D, E, F, G, H, I, J, and $\gamma$. So the representations of the solutions and their stability are obtained in Figs. 2, 3, 4 and 5. Where we denote $\varphi _{{an}}$ the analytical solution $\varphi$ and $\varphi _{{nu}}$ the numerical solution $\varphi$ and so on for the other solution $\xi$.

## Conclusion

The problem of pendulum motion with a suspended point at infinity is studied. We achieve a large parameter proportional to the radius of the circle which is sufficiently large. The approximated periodic solutions are obtained up to the third-order applying the method of large parameters^28^. For the non-resonance case, the quantities of the angular velocities $\Omega$ and $\sigma$ must have a non-integer value to avoid the singularities in the solutions. The analytical solutions for the considered problems are obtained analytically. These solutions are worked out by computer programs to get their graphical representations and to show the effect of increasing the angular velocity $\omega$. We deduce that when $\omega$ increases the solutions are stable and the stability diagrams take the cardioid forms and when $\omega$ decreases the stability diagrams take the ellipse forms. When b increases and $(a/b) \succ \succ 1$, the motion becomes more stable, see Figs. 4 and 5.

The obtained solutions (32, 33) describe the influence of the different parameters of the problem on the behavior of the motion. We conclude that when the spring's length is sufficiently large, the elastic constant $k$ must be nearer to the constant angular velocity $\omega$ of the pendulum motion. Also, we conclude that the case of the simple pendulum is obtained as a special case from this work when the sphere tends to a point ($a \to 0$) and the length of the spring tends to the length of the string ($\rho \to b$). We conclude also that the large parameter method solves the problem when the suspended point near infinity and the small parameter one solves it when the suspended point near zero.

As an application of this study is Schuler's pendulum^29–31^. This pendulum is suspended vertically such that it remains aligned to the local vertical even if its suspended point is accelerated parallel to Earth’s surface. This principle of such a pendulum is applied in some inertial guidance systems to maintain a correct internal vertical reference, even during rapid acceleration. Other applications are the Foucault and ballistic pendulum which are used to show the Earth's rotation about its axis.
